# STOC Free: An Innovative Framework to Compare Probability of Freedom From Infection in Heterogeneous Control Programmes

**DOI:** 10.3389/fvets.2019.00133

**Published:** 2019-04-26

**Authors:** Annika M. van Roon, Inge M. G. A. Santman-Berends, David Graham, Simon J. More, Mirjam Nielen, Aurélien Madouasse, Mathilde Mercat, Christine Fourichon, Jörn Gethmann, Jenny Frössling, Ann Lindberg, Carla Correia-Gomes, George J. Gunn, Carola Sauter-Louis, Madeleine K. Henry, Linda van Duijn, Gerdien van Schaik

**Affiliations:** ^1^Department of Farm Animal Health, Faculty of Veterinary Medicine, Utrecht University, Utrecht, Netherlands; ^2^Department of Epidemiology, GD Animal Health, Deventer, Netherlands; ^3^Animal Health Ireland, Carrick-on-Shannon, Ireland; ^4^Centre for Veterinary Epidemiology and Risk Analysis, Veterinary Sciences Centre, University College Dublin, Dublin, Ireland; ^5^BIOEPAR, INRA, Oniris, Université Bretagne Loire, Nantes, France; ^6^Friedrich-Loeffler-Institut, Institute of Epidemiology, Greifswald, Germany; ^7^Swedish National Veterinary Institute (SVA), Uppsala, Sweden; ^8^SRUC (Scotland's Rural College), Edinburgh, United Kingdom

**Keywords:** control programmes, output-based, freedom from disease, bovine viral diarrhea virus, bayesian statistics

## Abstract

The existence, stage of eradication and design of control programmes (CPs) for diseases that are not regulated by the EU differ between Member States. When freedom from infection is reached or being pursued, safe trade is essential to protect or reach that status. The aim of STOC free, a collaborative project between six countries, is to develop and validate a framework that enables a transparent and standardized comparison of confidence of freedom for CPs across herds, regions or countries. The framework consists of a model combined with a tool to facilitate the collection of the necessary parameters. All relevant actions taken in a CP are included in a Bayesian network model, which allows prior distributions for most parameters. In addition, frequency of occurrence and risk estimates for factors that influence either the probability of introduction or temporary misclassification leading to delayed detection of the infection are included in the model. Bovine viral diarrhea virus (BVDV) is used as an example disease. Many countries have CPs in place for BVDV and although elements of the CPs are similar, biosecurity measures and testing protocols, including types of tests and testing frequency, as well as target groups, differ widely. Although the initially developed framework is based on BVDV, the aim is to make it sufficiently generic to be adaptable to CPs for other diseases and possibly other species. Thus, STOC free will result in a single general framework, adaptable to multiple disease CPs, which aims to enhance the safety of trade.

## Introduction

Several European countries have implemented national or regional surveillance, control, or eradication programmes for non-regulated infections of cattle, such as bovine viral diarrhea (BVDV), paratuberculosis and salmonellosis. These programmes bring tangible benefits to participating farmers and national economies and are to be strongly supported. However, they also create difficulties for intra-community trade as free trade between European countries has the potential to allow the movement of infectious agents into regions where freedom from infection has been achieved (1–3). Control programmes (CPs) in European countries generally differ in the way that the free status is achieved and assigned, which makes it difficult to assess whether confidence of freedom from infection (the output) is equivalent. An understanding of equivalence with respect to freedom from infection is important when seeking to facilitate intra-community animal movements, whilst also managing the risk of infection. Up to now, there is a lack of agreed methodologies to assess and compare confidence of freedom from infection of cattle that are being moved between EU countries with different CPs.

There is currently minimal regulation at European level to control the spread of many important endemic diseases, including BVDV, between EU member states through the movement of animals. Therefore, there is a need for a tool that enables transparent and standardized comparison of confidence of freedom resulting from different CPs to facilitate safe trade. This tool should be able to calculate the confidence that animals moved between regions or countries are truly free from infection to prevent (re-)introduction of the infection in a free herd and/or territory. As there are many different CPs in place in different European regions and/or countries for non-regulated infections, there is an increasing need to implement output-based standards for animal health surveillance (4–8). With output-based standards, the emphasis is placed on comparability of the required outcome i.e., confidence of freedom from infection and its associated uncertainty, and not on the processes required to achieve this outcome, i.e., input-based standards. A growing body of scientific literature supports the development of output-based standards in animal health (4–8). Several methods have been developed to calculate freedom of infection, including scenario tree models and Bayesian methods where multiple surveillance components are combined, and latent class methods that take time since sampling into account (9, 10). These methods are promising, but further research is need to allow simple and practical field-based application to enable standardized and quantitative comparison of outputs of CPs. A practical tool is needed to support the livestock industry in controlling and/or eradicating livestock infections.

## STOC Free

In 2017, a project was initiated by eight parties from six different countries (DE, FR, IE, NL, SE, UK) to develop and validate a Surveillance analysis Tool for Outcome-based Comparison of the confidence of FREEdom (STOC free) resulting from different control or eradication programmes. The STOC free framework fulfills the need to implement output-based standards for control of cattle infections by development of a single general output-based framework. The STOC free framework provides an objective and uniform approach to assess the probability of freedom from infection and its associated uncertainty given the heterogeneity in context and design of the CP.

**Figure 1:**
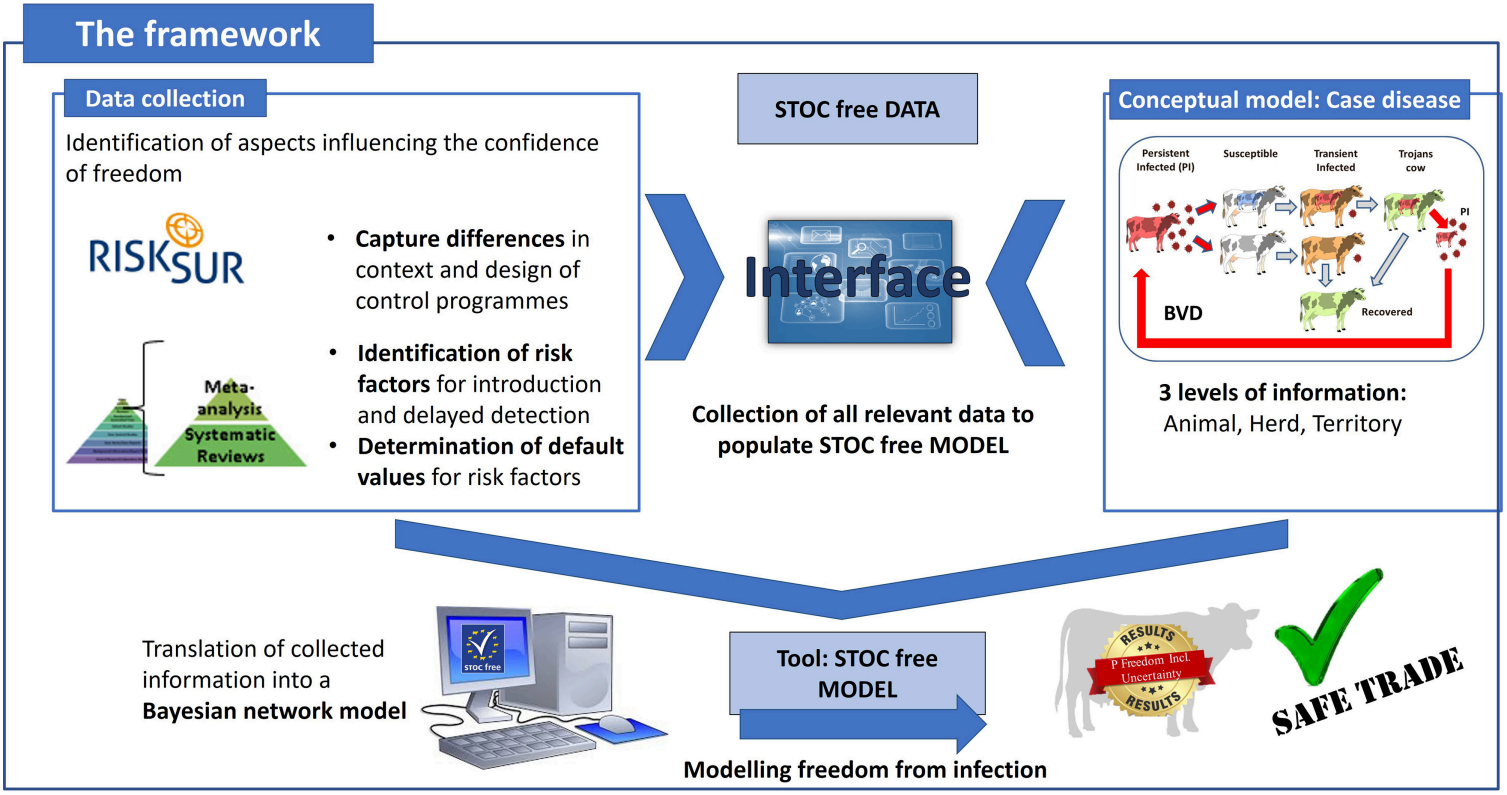
Graphical representation of the STOC free framework consisting of a data collection tool (STOC free DATA) and a model for calculation of freedom from infection (STOC free MODEL).

The developed framework consists of a model (STOC free MODEL) combined with a tool to facilitate the collection of the necessary quantitative input information (STOC free DATA). To support the development of STOC free DATA and STOC free MODEL, a case disease was first chosen to use as example disease, i.e., BVDV. Detailed information about the different CPs for BVDV in the six partner countries was collected with the RISKSUR tool (the RISKSUR tool, http://www.fp7-risksur.eu/results/tools). Information on risk factors for introduction and delayed detection of BVDV was collected by performing a systematic review, and default values for STOC free model were generated by meta analyses. With a conceptual model, the infectious process of BVDV within the animal and transmission of BVDV within and between herds was described to fully understand the dynamics of infection and decide on the type of model that best suited the STOC free aim (see Figure 1).

## Example Disease: Bovine Viral Diarrhea Virus

For development and evaluation of the STOC free framework, BVD is used as an example disease. In Europe, many countries have differently designed CPs in place for BVDV and are at different stages of eradication, ranging from endemic infection to freedom. BVDV was specifically chosen as the model disease, because of the differences in infectiousness between transiently and persistently infected (TI, PI) cattle (11–13) and the occurrence of both horizontal and vertical transmission. Horizontal transmission results in TI cattle, which are viraemic for a short period of time after which they recover and become immune for life. Vertical transmission during early gestation can result in the birth of PI calves. PI animals spread virus in large quantities throughout their lifetime and thus are the most important source for spread of the virus (14). BVDV is often introduced by purchase of either PI animals or cows pregnant with PI calves. The latter are commonly referred to as Trojan cows due to the hidden way in which such cows can introduce the virus into a new herd.

## Development of STOC Free Data

The STOC free data tool (STOC free DATA) is designed to guide the user of the STOC free framework to gather the information and data needed to populate the STOC free MODEL. As a first step in the development of STOC free DATA, BVDV control programmes in place in the countries of the STOC free partners were described in a very detailed way. All aspects related to BVDV and the CPs in place in the participating partner countries were collected using an existing tool for harmonized description of surveillance programmes (the RISKSUR tool, http://www.fp7-risksur.eu/results/tools). This tool was originally developed to describe and (re-)design single surveillance components and did not meet all the criteria for application to BVDV CPs. Therefore, the RISKSUR tool was expanded to also gather information on the control actions described in the BVDV CPs and country-specific risk factors for introduction of BVDV and delayed detection. Following completion of the data collection through the RISKSUR tool by all collaborating countries, it was possible to list those variables that differed substantially between CPs and could potentially lead to variation in confidence of freedom and associated uncertainty (Table 1).

**Table 1:** Comparison of BVD control programmes and BVD status in six European countries in 2017.

**Elements**	**Countries**					
	**DE**	**FR (Brittany)**	**IE**	**NL**	**SE**	**UK (Scotland)**
Herd level prevalence (breeding herds)	0.08%	unknown	2%	9%	0%–free	10%
Type of programme	Mandatory	Voluntary	Mandatory	Voluntary	Mandatory	Mandatory
Type of testing–screening/case finding	Ear notch, blood/serum	Bulk milk, ear notch, blood/serum	Ear notch	Bulk milk, ear notch, blood/serum	–	Ear notch, blood/serum
Type of testing - monitoring freedom of disease	Ear notch, blood/serum	Bulk milk, ear notch, blood/serum	Ear notch	Ear notch, blood/serum	Bulk milk, blood/serum	Blood/serum
Vaccines licensed for use	Yes	Yes	Yes	Yes	No	Yes
Funding	Private and public	Private	Private and public	Private	Private and public	Private
Most important herd level risk factors for introduction:						
1	Introduction of imported cattle	Boundary contact with neighboring cattle herds	Boundary contact with neighboring cattle herds	High cattle density	Introduction of imported cattle	Delayed removal of known PI animal(s)
2	Introduction of TI cattle	Introduction of cattle	Introduction of pregnant cattle	Introduction of pregnant cattle	–	Introduction of cattle with unknown status
3	Introduction of pregnant cattle	Presence of fattening unit	Indirect transmission through personnel	Indirect transmission through professional visitors	–	Boundary contact with neighboring cattle herds

*DE, Germany; FR, France; IE, Ireland; NL, Netherlands; SE, Sweden; UK, United Kingdom*.

Differences and similarities between CPs were captured to identify aspects that can directly or indirectly influence the confidence of freedom from infection in a BVDV CP. The probability and associated uncertainty that an animal from a herd declared free by a given CP is truly free from infection at the moment of trade is influenced by the risk that:

- the infection was (re-)introduced into the herd after the last round of testing i.e., risk of introduction, or
- the latest round of testing resulted in a false negative result in relation to the herd's true positive status, i.e., misclassification leading to delayed detection either of newly introduced, or residual infection.

The risk of introduction and delayed detection are influenced by the control measures in place in CPs but also by the existence and relative importance of country-specific risk factors. For BVDV, the most important risk factors were identified by STOC free partners as communal grazing, trade of live cattle and cattle density, i.e., number of cattle per km^2^, the latter being considered to be a proxy for the number of neighboring herds with which contact can potentially occur via direct or indirect transmission pathways.

Additionally, a systematic review of risk factors described in relevant scientific literature was conducted to obtain a comprehensive overview of all aspects that could influence either the probability of introduction of BVDV or could result in misclassification leading to delayed detection of the virus. Using meta analyses, we aim to determine generic risk estimates for the most influential risk factors for introduction or delayed detection that can be used as default values in the STOC free framework.

Data availability, quality and format were evaluated per country. Variables could only be included in the framework when at least some countries had quantitative data available for the respective variable. Variables for which data are lacking in some countries could still be included in the model when deemed important. Such variables would be included using default values for countries in which quantitative data was not available. The defaults can be replaced by more precise estimates once data becomes available; thereby “future-proofing” the framework.

## Modeling Freedom of Infection

An essential step in the development of the STOC free framework was the design of a conceptual model representing the infectious process of BVDV at different levels, from animal to region. The conceptual model consisted of diagrams and explanatory text and maps the different types of information related to BVDV influencing the true status regarding infection. At the individual animal level, this included the different epidemiological states such as PI, TI, immune post infection, and susceptible, the course of infection and diagnostic results. The conceptual model at the herd level presented within herd infection dynamics, including risk factors for introduction and testing strategies employed. The conceptual model at territory level mapped the between herd infection dynamics, including contact structure both within and between territories and prevalence. Based on the information of the conceptual model and discussions among the partners, it was decided that the final STOC free MODEL should:

- Include informative priors and temporal aspects
- Allow input and output distributions to include biological variation and uncertainty
- Provide a generic probability and related uncertainty when no specific information is present, becoming more specific for individual situations by adapting the default information in STOC free DATA.
- Provide confidence in the free status of an animal at the moment of leaving the farm

Currently, the information resulting from the conceptual model is being translated into a Bayesian network model (STOC free MODEL). Bayesian networks are flexible and allow structuring heterogeneous information for the estimation of a uniform output. Within the STOC free project, the Bayesian network will be represented using directed acyclic graphs (DAG). Each node on the network represents a parameter that influences the probability or confidence of freedom from BVDV infection and is expressed by means of a statistical distribution. Each node in the DAG is connected to one or more nodes through arrows. For example, a node herd BVD status with a Bernoulli distribution can be connected to a node bulk tank milk ELISA optical density with a Normal distribution. In this case, the value of the ELISA test result can be modeled as a function of the BVD status. Given the heterogeneity in CPs, data will be available for some of the nodes and missing for others e.g., bulk tank milk ELISA available, calf ear notch antigen test missing. Available data will be used to estimate the parameters of the statistical distributions and allow a distribution for missing data to be provided. In all cases, the distribution of the probability of freedom from infection will be the quantity of interest and will be estimated from all the available data.

## Validation and Wider Application of the Framework

The developed framework will be tested and validated using case studies to evaluate the probability of freedom from BVDV infection on animal, herd and territory level in each of the collaborating countries in which the BVDV situation varies from endemic to free. Application of the framework will result in a numerical and objective evaluation of CPs for BVDV in the EU. Transfer of this knowledge will enable countries to learn from each other, to optimize existing CPs and to improve the design of CPs for other diseases. Although BVDV will provide a rigorous test of the flexibility of the framework as initially developed, the framework should be generic enough to be adaptable to CPs for other diseases. At a later stage of the project, the possibilities for expanding the framework to other diseases and other species will be explored.

## Limitations of the Framework

The STOC free framework is first developed for BVDV in cattle. Currently, it is not yet applicable to other pathogens or other animal species. Within the STOC free project, the potential for expansion of the framework will be explored. There is currently no socioeconomic information incorporated in the model. At a later stage, it would be beneficial to include such information noting that CPs could generate a very high confidence of freedom, however, this may be achieved in a manner that is not cost-effective. Also social aspects should be taken into account. For example, stamping out could be very cost-effective and the fastest way to eradicate infection, but is not always easily accepted by the community. Incorporating these factors into the model are foreseen as next step in the development of a sustainable STOC free framework.

## Vision

The ultimate goal is that the STOC free framework can estimate the probability of freedom from BVDV infection and the uncertainty around that probability for a traded animal from a free herd or region in a given CP and that it will be used throughout Europe to enhance safe trade. The framework can be used by organizations with access to the required data and good understanding of the disease control programmes. The process will be supported by a COST Action SOUND control (CA17110) in which a large number of participants from many European countries are involved. The COST action aims to coordinate, stimulate and assist initiatives to explore and implement a widely adaptable output-based framework. The long-term vision is that the framework will be used by European countries to objectively assess equivalence in the probability of freedom of traded animals for any infectious disease given differently designed CPs tailored to the unique demographic situation of each specific country.

## Author Contributions

The manuscript results from a project called STOC free which is coordinated and presented at InnovSur by GvS. AvR prepared the manuscript, which was revised primarily by GvS, IS-B, MN, DG, SM, AM, JG, AL, JF, and C-CG. Conceptual contributions were made by CF, MM, CS-L, GG, LvD, and MH.

### Conflict of Interest Statement

The authors declare that the research was conducted in the absence of any commercial or financial relationships that could be construed as a potential conflict of interest.
